# Robotic Beyond Total Mesorectal Excision (bTME) for locally advanced and recurrent anorectal cancer: a systematic review

**DOI:** 10.1007/s11701-025-02573-1

**Published:** 2025-07-16

**Authors:** Joachim Cheng En Ho, Aryan Raj Goel, Muriel Sirgi, Ayan Bin Rafaih, Ayaz Ahmed Memon, Irshad Shaikh, Muhammad Rafaih Iqbal

**Affiliations:** 1 UCL Medical School, London, UK; 2 University Hospital Sussex NHS Foundation Trust, Worthing Hospital, Worthing, UK; 3 Aitchison College, Lahore, Pakistan; 4 Royal Liverpool University Hospital, Liverpool, UK; 5 Norfolk and Norwich University Hospitals NHS Foundation Trust, Norwich, UK; 6 University of East Anglia, Norwich, UK

**Keywords:** Robotic surgery, Minimally invasive surgery, Anorectal cancer, Beyond total mesorectal excision, Pelvic exenteration

## Abstract

Robotic surgery is increasingly utilized for rectal cancer resection, particularly in cases requiring beyond total mesorectal excision (bTME) to achieve oncological clearance. Despite longer operative times, robotic bTME has been associated with reduced morbidity and blood loss, making it an emerging approach in specialized centers. A systematic review following PRISMA guidelines was conducted in Web of Science, PubMed, and Scopus. Studies reporting robotic bTME for recurrent or locally advanced anorectal cancer were included. Outcomes assessed included study characteristics, demographics, operative outcomes, oncological data, and follow-up. Nineteen studies comprising 1027 patients met the inclusion criteria (13 case series—68% and 6 cohort studies—32%). The median patient age ranged from 51 to 68 years with 73.7% males. Most patients had an ASA score of 2 (53.1%), and BMI ranged from 21.1 to 28.6. Tumor locations were predominantly near the anal verge (median: 3–6 cm), and the most common clinical staging was cT3, cN1, and cM0. Surgical complications included urinary issues (22.6%), anastomotic leakage (11.4%), ileus (10.4%), and bleeding (5.3%). Follow-up data indicated a recurrence rate of 24.9%, and the 1-year survival rate was > 90%. These studies reported an overall complication rate of 49.7%, with a median follow-up of 12–36 months. Oncological outcomes were favorable, although there was significant variability in survival data between studies. The heterogenicity of the studies makes it challenging to conclusively establish robotic bTME as a feasible alternative to the gold standard. Further prospective studies, with measurable outcomes and consistent terminology, are needed to ensure homogeneity.

## Introduction

Total Mesorectal Excision (TME) for rectal cancer was described by Heald in 1982 and is the gold standard for rectal cancer surgery [1]. It is based on the principle of dissection in the embryological planes thus excising the rectum along with the mesorectum. This has resulted in the reduction in 5-year local recurrence rate after TME to 4% [2]. Rectal cancer surgery has evolved in the past decade with the advancements in the neoadjuvant therapy and increasing use of minimally invasive approaches. Robotic surgery is increasingly being utilized for rectal cancer surgery owing to its benefits of reduced length of hospital stay, faster recovery and reduced complications [2].

About 10–20% of the patients present with locally advanced rectal cancer (LARC) with involvement of the adjacent organs and lateral pelvic lymph nodes [3, 4]. These patients require combination of neoadjuvant chemoradiotherapy and Beyond TME (bTME) to achieve R0 resection which is directly related to recurrence and overall survival [5]. bTME is an umbrella term encompassing three types of approaches described as “three degrees of dissection” [6]. This includes radial (adjacent pelvic organs), lateral (pelvic lymph node involvement) and distally longitudinal (intersphincteric resection and abdominoperineal resection) [6, 7]. bTME includes pelvic exenteration which involves the removal of all or most pelvic organs. There is considerable heterogenicity between parameters used to describe pelvic exenteration. Burns et al. [8] set out to create a lexicon to allow for standardization in reporting for this complex surgery.

Most of the bTME procedures are performed with open approach. Selected patients in specific centers do undergo laparoscopic bTME depending upon the extent of resection owing to the limitation of straight laparoscopic instruments [9, 10]. The first report of robotic pelvic exenteration for locally advanced rectal cancer was by Shin et al. [11] in 2014. Robotic surgery is used in specialized centers around the world for bTME although the adoption is slow due to the technically challenging oncological resections with high intraoperative complications. Theoretically robotic surgery offers the advantage of stable 3D camera, visual magnification, endo-wrist instruments and advanced ergonomics in the narrow confinements of the pelvis [12, 13]. There have been comparisons of laparoscopic and robotic bTME with the latter providing favorable short-term outcomes such as lower estimated blood loss and lower incidence of urinary retention alongside smaller incidence of postoperative urinary function impairment [14] with very small data on oncological survival [15–17].

The authors aim to look at the outcomes for robotic bTME for locally advanced and recurrent ano-rectal cancers.

## Materials and methods

The present systematic review was carried out and reported according to the Preferred Reporting Items for Systematic Reviews and Meta-Analyses (PRISMA) statement.

### Search strategy

The protocol was registered in the International Prospective Register of Systematic Reviews (PROSPERO) with the registration number CRD42024576046 in August 2024. The authors performed a systematic literature search through Web of Science, PubMed and Scopus for articles up to 5 August 2024. The keyword searches were carried out with specific automated filters outlined in the exclusion criteria below (MeSH Terms were only used in PubMed and were swapped out for their non-MeSH counterparts in Web of Science and Scopus since they were unsupported) (Table 1).

**Table 1:** Keyword search

No	Query
1	“robot*” OR “robotic surgical procedures” [MeSH Terms]
2	“pelvic exenteration*” OR “pelvic evisceration*” OR “pelvic resect*” OR “pelvic exenteration” [MeSH Terms] OR “beyond total mesorectal excision*”
3	“recurren*” OR “advance*”
4	“rectal” OR “anal”
5	“neoplasm*” OR “malignan*” OR “cancer*” OR “tumor*”
6	#1 AND #2 AND #3 AND #4 AND #5
Automated filters used	All: Title and Abstract searches; Web of Science: articles and early access, English-only; Scopus: articles, English-only

### Inclusion and exclusion criteria

#### Inclusion criteria

1. Studies reporting locally advanced or recurrent anorectal cancer outcomes following robotic bTME
2. Studies comparing robotic and laparoscopic bTME where data for robotic bTME can be extracted (> 50%)

#### Exclusion criteria

1. Studies without full text
2. Letters, commentaries, short communications, conference abstracts, editorials and videos
3. Case series with fewer than five patients
4. Studies where < 50% of the sample size with extractable data specifically underwent robotic bTME procedures for anorectal cancers.
5. Studies with overlapping sample sizes, in which the study with the larger sample size will be included.

Several studies were excluded based on the predefined criteria to ensure data specificity and avoid duplication. Gomez Ruiz et al. [18] did not meet exclusion criterion (4), as only six out of 41 patients underwent robotic bTME, and no subgroup data were presented for these patients. Regarding exclusion criterion (5), Lokuhetty et al. [19] and Larach et al. [20] appeared to report overlapping cohorts; the latter study, which included a larger sample (n = 24 vs. n = 8), was retained. Similarly, two additional studies [14, 21] shared a first author and overlapping patient population with Kim et al. [22]; therefore, the study with the largest sample size (n = 100) was included over the others (n = 50 and n = 11).

J.C.E.H. and A.R.G. independently screened through the titles, abstracts, and full-text articles against the eligibility criteria after reviewing them on an online Excel sheet. M.R.I. was responsible of solving any disagreements.

### Outcome measurements

Outcomes reported were separated into five main categories:

1. Study characteristics: first authors, years of publication, the country where the study was carried out, sample size and study design.
2. Patient baseline characteristics and clinical data: age, gender, body mass index (BMI) and American Society of Anesthesiologists (ASA) ratings, tumor location and clinical stage including specific TNM staging where applicable.
3. Operative outcomes: type of bTME procedure performed, exclusion criteria, operation duration, estimated blood loss (EBL), complication rate (including complication details), Clavien-Dindo (CD) grade, length of stays (LOS), recorded areas of resections, total lymph nodes harvested, conversions, reoperations and readmissions.
4. Oncological outcomes: resection margin (R) status and/or circumferential resection margin (CRM) status, pathological staging, details on neoadjuvant and adjuvant therapies and mortality.
5. Follow-up data: duration, overall recurrence, local recurrence, distant recurrence, disease-free survival and overall survival.

### Quality assessment

The studies were assessed based on their comparability. The studies consisted of 13 case series (as defined previously as ≥ 5 cases) and six cohort studies. Case series were assessed using the JBI Critical Appraisal Tool [23] and Cohort studies were assessed using the Modified Newcastle–Ottawa Score for non-randomized studies [24]. This was carried out by the two authors J.C.E.H. and A.R.G. independently and disagreements were resolved by M.R.I.

## Results

### Included studies

**Fig. 1:**
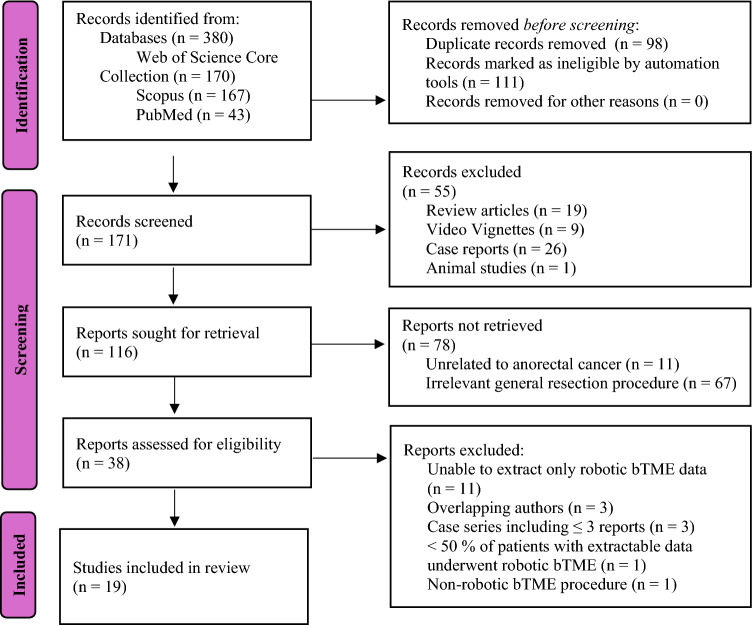
Summary of the systematic search in accordance with PRISMA guidelines

The PRISMA flow diagram below summarizes the results of the search (Fig. 1). Web of Science Core Collection, Scopus and PubMed retrieved 170, 167 and 43 articles respectively. Of the 380 articles, 98 duplicates were manually removed, and 111 articles were removed by the automated filters (111 non-English articles, of which 98 were also marked as non-articles). Manual first pass screening excluded 55 articles and full text screening removed another 19 articles. 19 studies were included in the final analysis.

### Study characteristics

Of the 19 included studies, 13 (68%) and 6 (32%) of them were case series and cohort studies respectively. All included studies were retrospective, published between 2016 to 2024 and had varying sample sizes (5–168), with a total of 1027 patients analyzed in this review. These studies were from USA (n = 5, 26%), South Korea (n = 4, 21%), UK (n = 3, 16%), Japan (n = 4, 21%), India (n = 3, 16%), France (n = 1, 5%), Ireland (n = 1, 5%), Turkey (n = 1, 5%), Italy (n = 1, 5%), Norway (n = 1, 5%) and Australia (n = 1, 5%) (Table 2).

**Table 2:** Study characteristics of included articles

First author	Study design	Year of publication	Country	Sample size
Chang et al. [25]	Retrospective Case series	2023	UK	17^a^
Dibrito et al. [26]	Retrospective Case series	2023	USA	86
Ishizaki et al. [27]	Retrospective Cohort study	2023	Japan	27
Jaganmurugan et al. [28]	Retrospective Case series	2022	India	5
Kazi et al. [29]	Retrospective Cohort study	2023	India	74
Khan et al. [7]	Retrospective Case series	2024	France, India, Ireland, South Korea, Turkey, UK, USA	168
Kim et al. [22]	Retrospective Cohort study	2022	South Korea	100
Larach et al. [20]	Retrospective Case series	2022	Australia	24^b^
Peacock et al. [30]	Retrospective Case series	2020	USA	40^c^
Piozzi et al. [6]	Retrospective Case series	2020	Italy, South Korea	137
Saqib et al. [31]	Retrospective Case series	2024	UK	13^d^
Shin et al. [32]	Retrospective Case series	2016	USA	36
Sieffert et al. [33]	Retrospective Case series	2016	USA	6
Thorgersen et al. [34]	Retrospective Case series	2023	Norway	105
Tokunaga et al. [35]	Retrospective Cohort study	2024	Japan	19
Williams et al. [36]	Retrospective Case series	2020	Australia	5^e^
Yamaguchi et al. [37]	Retrospective Cohort study	2018	Japan	78^f^
Yang et al. [38]	Retrospective Case series	2021	South Korea	23
Yatabe et al. [39]	Retrospective Cohort study	2024	Japan	59^ g^

^a^7 patients had their rectal resections carried out prior

^b^21 patients had primary/recurrent rectal adenocarcinoma, 1 had anal squamous cell and 2 had leiomyosarcoma carcinoma

^c^4 patients had their rectal resections carried out prior

^d^4 patients had primary gynecological malignancy

^e^2 patients had primary prostate cancer, 3 patients had synchronous prostate and rectal cancer

^f^Only data from the matched cohort was used in this review

^g^≤ 6 patients had bladder, prostate or ureteric primary cancer; Only data from the RPE vs. LPE propensity match scored group data was used in this review

### Patient characteristics

Age was reported in all 19 studies, though the statistical formats varied, including combinations of medians, means, interquartile ranges (IQR), ranges, and standard deviations (SD). Median age, reported in 15 studies [6, 7, 20, 22, 25–29, 32, 34–37, 39], ranged from 51 to 68 years. The overall mean age from four studies was 61.3 years [30, 31, 33, 39]. Reported IQRs commonly overlapped between 50 and 60 years [6, 7, 20, 26, 28, 31, 32, 36, 39], with the widest range being 44–71 years in one study [6]. Six studies reported cumulative age ranges spanning from 27 to 85 years [25, 27, 33–35]. Gender was described in all but one study [30], with males comprising the majority in 14 studies (73.7%) [6, 22, 25–27, 29, 30, 32–37, 39].

BMI was reported using different measures. Median values ranged between 21.1–28.6 kg/m^2^ [6, 7, 20, 26, 29, 30, 36, 39], while means ranged from 22.7–27.1 kg/m^2^ [22, 31, 33, 38]. The narrowest and widest IQRs were 24.4–27.7 kg/m^2^ [7] and 20.15–28.35 kg/m^2^ [36], respectively. Six studies reported BMI ranges spanning from 16.7–40.8 kg/m^2^ [25, 27, 33–35, 37]. One study [28] did not report BMI data, and another [32] identified BMI ≥ 30 kg/m^2^ without providing summary statistics.

ASA classification was described in 15 studies [6, 7, 20, 22, 25–27, 29–32, 34, 36–39], of which 13 reported distributions for individual scores [6, 7, 20, 22, 26, 27, 29–32, 34, 36–38]. Across these, 182 (20.5%) patients were ASA 1, 471 (53.1%) ASA 2, 202 (22.8%) ASA 3, 10 (1.1%) ASA 4, and 22 (2.5%) had missing ASA data.

Tumor location was commonly reported by distance from the anal verge [6, 22, 25, 27, 30, 36–38], and overall, 14 studies described tumor height [6, 20, 22, 25–27, 29–32, 35–38]. Median distances ranged from 3–6 cm [6, 25, 27, 30, 36, 37], with cumulative ranges of 0–10 cm in four studies [25, 27, 36, 37]. Four studies described rectal subsite (upper, middle, lower), with 60.7% located in the lower rectum [20, 22, 29, 32].

Primary cancers were reported in 13 studies [6, 20, 25–32, 34, 37, 39], of which five also included recurrent cancers [20, 25, 26, 31, 39]. Clinical staging was most often described using the TNM system [6, 7, 20, 26–30, 32, 34–37, 39], with cT3 (n = 435, 51.3%), cN1 (n = 285, 38.5%), and cM0 (n = 528, 83.3%) being the most reported individual categories. Five studies provided overall TNM stage groupings [7, 20, 22, 32, 35], with stage III (n = 180, 52.9%) being most common, followed by stage II (n = 73, 21.5%) and stage IV (n = 29, 8.5%) (Table 3).

**Table 3:** Patient characteristics

First author		Age (median, IQR)	Male sex (n, %)	BMI, in kg/m^2^ (median, IQR)	ASA score (n, %)	Tumor distance from AV, in cm (median, IQR or n, %)	Primary ± recurrent ArCa (n, %)	Clinical staging			
								cT (n, %)	cN (n, %)	cM (n, %)	Overall (n, %)
Chang et al. [25]		67, 27–79^a^	12, 70.6	26, 20–40^a^	2^f^, 2–3^a^	6, 4–10^a^	Both	NA	NA	NA	NA
Dibrito et al. [26]		55, 42–62	62, 72.1	26.5, 23.1–30.6	2: 12, 14 3: 71, 82.6 4: 3, 5	RSC: 4, 4.7 Rm: 67, 77.9	Both	Tx: 3, 3.5 T2: 3, 3.5 T3: 40, 46.5 T4: 40, 46.5	Nx: 2, 2.3 N0: 13, 15.1 N1: 27, 31.4 N2: 41, 47.7 N+; 3, 3.5	M0: 74, 86 M1: 12, 14	NA
Ishizaki et al. [27]		61, 42–80^a^	20, 74.1	21.1, 15.6–30.5^a^	1: 11, 40.7 2: 16, 59.3	4, 2–10^a^	PC	T3: 17, 63 T4: 10, 37	N1: 17, 63 N2: 10, 37	NA	NA
Jaganmurugan et al. [28]		53, 42.5–66	NA	NA	NA	NA	PC	T3: 1, 20 T4: 2, 40 T4b: 2, 40	N0: 1, 20 N1: 2, 40 N2: 1, 20 N2c: 1, 20	M0: 4, 80 M1: 1, 20	NA
Kazi et al. [29]		52, 36–60	55, 74.3	22.8, 20.1–26.2	1: 44, 59 2: 29, 39 3: 1, 1.4	*U*: 8, 11 *M*: 27, 36 *L*: 39, 53	PC	T2: 1, 1.4 T3: 31, 42 T4: 42, 57	N0: 6, 8.1 N1: 30, 41 N2: 38, 51	M0: 67, 91 M1: 7, 9.5	NA
Khan et al. [7]		60.0, 50.0–68.7	73, 43.2	24.0, 24.4–27.7*	1: 27, 16.0 2: 70, 41.4 3: 48, 28.4 4: 2, 1.2 missing: 22, 13.0	NA	NA	Tx: 6, 3.6 T1: 8, 4.8 T2: 16, 9.5 T3: 58, 34.5 T4: 80, 47.6	Nx: 7, 4.2 N0: 63, 37.5 N1: 58, 34.5 N2: 40, 23.8	M0: 154, 91.7 M1: 14, 8.3	0: 22, 13.1 I: 34, 20.2 II: 62, 36.9 III: 36, 21.4 IV: 14, 8.3
Kim et al. [22]		57.7, 11.3^c^	64, 64.0	23.9^b^, 2.8^c^	1: 36, 36 2: 57, 57 3: 7, 7	4.7^b^ ± 2.4^c^ *M*^*e*^: 33, 33.0 *L*: 67, 67.0	NA	NA	NA	NA	III: 93, 93.0 IV: 7, 7.0
Larach et al. [20]		59, 47–71.5	12, 50	26, 24.3–28.1	1: 4, 16.7 2: 11, 45.8 3: 9, 37.5	*U*: 3, 12.5 *M*^*e*^: 5, 20.8 *L*: 16, 66.7	PC: 17, 70.8 RC: 4, 16.7	T3a: 1, 5.9 T3b: 3, 17.6T3c: 1, 5.9 T4a: 1, 5.9 T4b: 11, 64.7	N0: 4, 23.5 N1: 3, 17.6 N2: 9, 52.9	NA	II: 4, 23.5 III: 13, 76.5
Peacock et al. [30]		54^b^, 15^c^	27, 67.5	28.6, 25.5–32.6	2: 5, 12.5 3: 3, 87.5	4, 3–7	PC	T3: 29, 80.6 T4: 7, 19.4	NA	NA	NA
Piozzi et al. [6]	Lateral	56, 49–60	15, 50	23.0, 20.1–25.2	1: 2, 6.7 2: 28, 93.3	4.5, 3.0–6.0	PC	T1: 1, 3.3 T2: 2, 6.7 T3: 22, 73.3 T4: 5, 16.7	N0: 4, 13.3 N1: 10, 33.3 N2: 16, 53.3	M0: 19, 63.3 M1: 11, 36.7	NA
	Longitudinal	57, 49–68	69, 78.4	23.8, 21.1–26.0	1: 25, 28.4 2: 61, 69.3 3: 2, 2.3	3.0, 2.5–3.0	PC	T1: 1, 1.1 T2: 10, 11.4 T3: 71, 80.7 T4: 6, 6.8	N0: 33, 37.5 N1: 37, 42.0 N2: 18, 20.5	M0: 77, 87.5 M1: 11, 12.5	NA
	Radial	51, 44–71	12, 63.2	22.5, 19.8–24.2	1: 5, 26.3 2: 14, 73.7	5.0, 2.2–8.0	PC	T3: 7, 36.8 T4: 12, 63.2	N0: 3, 15.8 N1: 9, 47.4 N2: 7, 36.8	M0: 13, 68.4 M1: 6, 31.6	NA
Saqib et al. [31]		60.4^b^, 53.6–67.2, 10.1^c^	0	27.1^b^. 23.7–30.5, 5^c^	2: 10, 76.9 3: 3, 23.1	Anal canal: 8, 61.5 Rm: 1, 7.7 Endometrium: 1, 7.7 Cervix: 2, 15.3 ≥ 1 organ: 1, 7.7	PC: 2, 15.4 RC: 11, 84.6	NA	NA	NA	NA
Shin et al. [32]		51, 46.5–60.5	22, 61.1	≥ 30: 12, 33.3	2: 10, 27.8 3: 25, 69.4 4: 1, 2.8	*U*: 6, 16.7 *M*: 10, 27.8 *L*: 20, 55.6	PC	T2: 3, 8.3 T3: 23, 63.9 T4: 10, 27.8	N0: 6, 16.7 N1: 18, 50 N2: 12, 33.3	NA	I: 2, 5.6 II: 4, 11.1 III: 24, 66.7 IV: 6, 16.7
Sieffert et al. [33]		63^b^, 47–72^a^, 9^c^	4, 66.7	26^b^, 17.6–34.4^a^, 6.7^c^	NA	NA	NA	NA	NA	NA	NA
Thorgersen et al. [34]		62, 28–85^a^	67, 63.8	25.9, 12.6–40.8^a^	2: 78, 74 3: 25, 24 4: 2, 2	NA	PC	T2: 2, 2 T3: 57, 54 T4b: 46, 44	N0: 24, 23 N1: 32 31 N2: 34, 32	M0: 70, 67 M1: 35, 33	NA
Tokunaga et al. [35]		68, 44–84^a^	14, 73.7	24, 19–28^a^	NA	PSJ: 3, 15 Rm^d^: 17, 85	NA	T2: 1, 5 T3: 12, 63 T4: 6, 32	N0: 3, 16 N1: 9, 47 N2: 4, 21 N3: 3, 16	NA	II: 3, 16 III: 14, 74 IV: 2, 11
Williams et al. [36]		64, 54–75.5	5, 100	23.8, 20.15–28.35	1: 2, 40 2: 2, 40 3: 1, 20	4, 1–7^a^	NA	T1: 1, 20 T2: 1, 20 T4: 1, 20 T4a: 1, 20 T4b: 1, 20	N0: 3, 60 N1: 1, 20 N2a: 1, 20	M0: 3, 60 M1: 2, 40	NA
Yamaguchi et al. [37]		63, 36–78^a^	58, 74.4	22.7, 16.7–29.7^a^	1: 23, 29.5 2: 51, 65.4 3: 4, 5.1	5.0, 0–8^a^	PC	T1: 1, 1.3 T3: 63, 80.8 T4: 14, 17.9	N0: 20, 25.6 N1: 32, 41 N2: 26, 33.3	NA	NA
Yang et al. [38]		55.3^b^, 11.2^c^	10, 43.5	22.7^b^, 3.8^c^	1: 3, 13 2: 17, 73.9 3: 3, 13	2.8^b^ ± 0.9^c^	NA	NA	NA	NA	NA
Yatabe et al. [39]		65, 57–74	51, 86.4	21.9, 19.9–23.2	≤ 2: 47, 80 ≥ 3:12, 20	NA	PC: 54, 92 RC: 5, 8	T2: 1, 2 T3: 4, 7 T4: 49, 91	N0: 15, 28 N ≥ 1: 39, 72	M0: 47, 87 M1: 7, 13	NA

Summary: Among the 19 studies, patients had a median age ranging from 51 to 68 years, with males forming the majority in 74% of studies. BMI varied widely, with medians between 21.1–28.6 kg/m^2^ and cumulative ranges from 16.7–40.8 kg/m^2^. ASA scores were predominantly ASA 2 (53.1%), followed by ASA 1 and 3. Tumor location was most often 3–6 cm from the anal verge, and 61% of tumors were in the lower rectum. Most tumors were primary and clinically staged as cT3, cN1, and cM0, with stage III being the most common overall classification

*IQR* interquartile range, *BMI* body mass index, *ASA* American Society of Anesthesiologists, *AV* anal verge, *ArCr* anorectal cancer, *NA* not available for retrieval, *PC* primary cancer, *RC* recurrent cancer,* RSC* rectosigmoid colon, *Rm* rectum, *U* upper rectum,* M* middle rectum, *L* lower rectum, *PSJ* proctosigmoid junction

^a^Range

^b^Mean

^c^Standard deviation

^d^Below the peritoneal reflection

^e^These studies defined upper rectum as > 10 cm; middle rectum as 6–10 cm; lower rectum as < 6 cm,

^f^Median

*Error in source data

### Surgical data

Exclusion criteria were described in 10 studies [7, 26–32, 38], commonly including palliative surgery, emergency resection, non-primary rectal cancer, non-en bloc adjacent organ resection, and distant metastasis. Operative duration was reported in all studies except three [28, 31, 37], most often as median with IQR (9 studies [6, 7, 19, 26, 29, 30, 32, 36, 39]) or as median with range (5 studies [25, 27, 33–35]); overall medians ranged from 280–690 min.

Estimated blood loss (EBL) was reported in all but one study [37], with median values ranging from 20–500 ml [7, 20, 25–36, 39], means of 125 ml [6, 22, 38], and a cumulative range of 0–2000 ml [6, 25, 27, 31, 33–35]. Complications were detailed individually or using CD grading in all but one study [37]. The most frequently reported complications were urinary issues (retention, leak, infection, dysfunction) [20, 22, 25, 27, 28, 30, 32, 35, 36, 38, 39], anastomotic leaks [6, 7, 20, 22, 27, 30, 38, 39], and ileus [6, 7, 20, 25, 27, 30, 36, 39]. Among the 14 studies reporting CD grades [6, 7, 20, 26–32, 35, 36, 38, 39], complications of CD ≤ II occurred in 34.9% (190/544, excluding [27, 35, 39]), while CD ≥ III complications were reported in 18.7% (129/689, excluding [27], using 30-day values from [39]).

Postoperative LOS was reported in 17 studies [6, 7, 20, 22, 25–33, 35, 36, 38, 39], with medians ranging from 4 to 25 days in 15 studies [6, 7, 20, 25–33, 35, 36, 39]; 8 studies [6, 7, 25, 26, 29, 30, 32, 36] reported median LOS < 10 days. The shortest and longest recorded stays were 3 days [25] and 63 days [31], respectively.

Organ resection results were inconsistently reported. The majority of studies [6, 20, 22, 25, 27, 29, 30, 34–38] used surgical classifications (e.g., APR, LAR, ISR), while two studies [7, 26] used a compartmental approach (anterior, posterior, lateral, central). Five studies [6, 20, 31, 36, 39] reported by pelvic exenteration type, while one focused solely on extralevator abdoperineal excision (ELAPE) [33]. The lateral compartment, often involving lateral pelvic node dissection (LPND), was the most consistently reported, appearing in 12 studies [7, 22, 25–30, 32, 34, 35, 37]; four specified laterality [25, 32, 35, 37].

Lymph node harvest was reported in 11 studies [7, 20, 22, 25–27, 29, 30, 32, 35, 37], with median counts ranging from 8–48; one study reported a mean of 19.6 nodes [22]. Among the 10 studies that reported conversion rates [6, 7, 19, 22, 25–27, 29, 30, 32], the overall rate was low at 1.8% (13/709). 30-day reoperation and readmission rates were 8.9% (37/414) and 15.9% (29/182), respectively [20, 25, 26, 28, 29, 31–35, 39] (Table 4).

**Table 4:** Surgical data

First author		Exclusion criteria	Operation duration, in min (median, IQR)	EBL, in ml (median, IQR)	Detailed complications (n, %)	CD (n, %)	LOS, in days (median, IQR)	Organs resected (n, %)	Total LN harvested (median, IQR)	Conversion rate (n, %)	30-day reoperation rate (n, %)	30-day readmission rate (n, %)
Chang et al. [25]		NA	280, 80–380^a^	20, 0–100^a^	LL: 1, 5.9 Ileus: 1, 5.9 UR: 1, 5.9	NA	7, 3–20^a^	TME + LPND: 10, 59 APER + LPND: 1, 6 LPND: 6, 35	8, 1–18^a^	0, 0	NA	2, 12
Dibrito et al. [26]		Non-en bloc resections; Extralevator abdominoperineal resections; Palliative resections; Non-colorectal cancer diagnoses	430, 367–606	175, 100–300	Overall: 33, 38.4 WD: 2, 2.3 Superficial SSI: 3, 3.5 Deep SSI: 4, 4.7 AL: 3, 3.5	≥ III: 10, 11.6	4, 3–7	Anterior: 62, 72.1 Posterior: 9, 10.4 Inferior: 29, 33.7 Lateral: 39, 45.3 (Bilateral: 11, 12.8; Unilateral: 13, 15.1)	26, 20–33	3, 3.5	4, 4.7	10, 11.6
Ishizaki et al. [27]		Multiorgan cancer; Deaths from another disease	117, 88–177^a^	113, 4–689^a^	Ileus: 2, 7.5 SSI: 2, 9.5 AL: 1, 3.7 LL: 1, 3.7 Urinary dysfunction: 3, 11.1	≥ II: 27, 100	14, 10–25^a^	LAR + LPND: 16, 59.2 ISR + LPND: 8, 29.6 APR + LPND: 3, 11.1	38, 5–76^a^	0, 0	NA	NA
Jaganmurugan et al. [28]		Primary/nodal disease with lateral extension that jeopardizes bladder vasculature bilaterally; Extension of disease into the bladder neck/trigone that threatens the ureteral orifices	NA	500, 400–1250	BO: 1, 20 UL: 1, 20	II: 2, 40 IIIb: 2, 40	14, 6–15	Bladder preserving PE: 5, 100 Additional Bilateral LPND: 1, 20	NA	NA	2, 40	NA
Kazi et al. [29]		Standard TME; Partial mesorectal excision	472, 335–600	400, 250–800	NA	0: 30, 41 I: 12, 16 II: 11, 15 IIIA: 9, 12 IIIB: 11, 15 IVa: 1, 1.4	8, 6–12	bTME-LAR/ISR: 25, 34 bTME-APR: 18, 24 PE: 31, 42 LPND: 41, 55 Multiple organs: 19, 26 Bladder/seminal vesicle/ prostate: 21, 28 Uterus/vagina: 10, 14 Soft tissue/bone: 4, 5.4	17, 11–24	0, 0	7, 9.5	NA
Khan et al. [7]		Palliative surgery; emergency procedures	314, 260–450	150, 27.5–500	AL: 14, 8.3 Ileus: 12, 7.1 Stoma complication: 5, 3.0 SSI: 14, 8.3	I–II: 47, 61.0 III: 27, 35.0 IV: 3. 3.9	8, 5–12	Anterior: 27, 16.1 Central: 137, 81.5 Posterior: 1, 0.6 LPND: 10, 7	17, 11–25	8, 4.8	NA	NA
Kim et al. [22]		NA	282.9^b^, 78.9^c^	82.7^b^, 103.6^c^	AL: 10, 10.0 Lymphocele: 6, 6.0 Leg pain or numbness: 2, 2.0 Leg oedema: 1, 1.0 Bleeding: 1, 1.0 Intra-abdominal abscess: 3, 3.0 Uncontrolled ascites: 2, 2.0 Wound infection: 1, 1.0 Rectovaginal fistula: 3, 3.0 Cardiac problem: 2, 2.0 Ureter stricture: 5, 5.0 UR: 4, 4.0	NA	10.1^**b**^, 8.0^c^	LAR: 50, 50 ISR: 45, 45 APR: 5, 5 LPND: Unilateral left: 39, 39; right: 31, 31; Bilateral: 30, 30	19.6^b^, 9.5^c^	0, 0	NA	NA
Larach et al. [20]		NA	380, 290–455	400, 200–2000	Postoperative fever (no source identified): 2, 8.3 Ileus: 3, 12.5 Pelvic collection: 3, 12.5 AL: 1, 9 Mechanical small BO: 2, 8.3 Flap dehiscence: 1, 4.2 UL/urosepsis: 1, 4.2	I: 1, 4.2 II: 5, 20.8 III: 8, 33.3	16, 9.3–23.8	TPE: 5, 20.8 LAR + AOR: 11, 45.8 APR + AOR: 8, 33.3	17, 11.5–21.5	1, 4.2	3, 12.5	3, 12.5
Peacock et al. [30]		Locally recurrent cancers at presentation; Non-curative treatment; Patients without post-nCRT imaging; Patients without NART	420, 313–540	150, 55–200	Overall: 15, 37.5 Ileus: 4, 10 UR: 6, 15 AL: 2, 5 PE: 1, 3 SVT: 1, 3 UTI: 2, 5 Perineal Wound infections: 2, 5	I: 4, 10 II: 10, 25 III: 4, 10	4, 3–6	LPND after rectal resection: 4, 10 Primary rectal resection + LPND: 36, 90 (actual operations LAR/APR not mentioned)	25, 17–34	0, 0	NA	NA
Piozzi et al. [6]	Lateral	NA	347, 301–410	67^b^, 0–500^a^	Overall: 16, 53.3 AL: 4, 13.3 Ileus: 6, 20.0	I–II: 10, 33.3 III–IV: 6, 20.0	9, 8–16	LAR + AOR: 18, 60 APE + AOR: 4, 13.3 ISR: 8, 26.7	NA	0, 0	NA	NA
	Longitudinal		300, 271–359	114^b^, 0–800^a^	Overall: 37, 42.0 AL: 5, 5.7 Ileus: 19, 21.6	I–II: 5, 5.7 III–IV: 14, 15.9	9, 7–13	ISR: 88, 100				
	Radial		415, 295–510	258^b^, 0–2000^a^	Overall: 14, 73.7 AL: 4, 21.1 Ileus: 7, 36.8	I–II: 7, 36.8 III–IV: 7, 36.8	17, 8–21	LAR + AOR: 6, 31.6 APE + AOR: 12, 63.1 Hartmann + AOR: 1, 5.3				
Saqib et al. [31]		Open/laparoscopic techniques; Benign pathology like complex diverticular disease	NA	200, 100–900^a^	NA	II: 5, 38.5 IIIA: 3, 23.1 IIIB: 1, 7.7	15, 7–63^a^	TPE: 3, 23 PPE: 10, 77	NA	NA	NA	6, 46.2
Shin et al. [32]		patients who could not be predicted to have complete resection of all disease based on multidisciplinary review of imaging	417.5, 337–496	200, 100–350	Deep pelvic abscess: 5, 13.9 Wound infection: 4, 11.1 WD (perineum): 1, 2.8 UTI: 3, 8.3 Hemorrhage: 1, 2.8 UR: 5, 13.9	I: 5, 13.9 II: 4, 11.1 III: 6, 16.7	4, 3–5.5	MVR: 22, 61.1 LPND: Unilateral: 9, 25; Bilateral: 3, 8.3 RPLND: 3, 8.3	20, 18–28	1, 2.8	4, 11.1	7, 19.4
Sieffert et al. [33]		NA	417, 305–495^a^, 66^c^	314, 150–400^a^, 105^c^	Perineal dehiscence: 1, 17 Donor site infection: 1, 17 Flap venous occlusion: 2, 33 Partial flap loss: 2, 33	NA	11.5, 6–20^a^, 4.6^c^	ELAPE: 6, 100	NA	NA	1, 17	1, 17
Thorgersen et al. [34]		NA	373, 235–636^a^	300, 50–1500^a^	Deep pelvic SSI: 21, 20 (Pelvic drain: 14, 67; Operative drainage: 4, 20) Perineal WD: 5, 5 Sepsis: 2, 2	NA	NA	APR: 63, 60 LAR: 8, 7.6 Hartmann: 34, 32.4 Anterior: 49, 46.7 Posterior: 27, 25.7 Lateral: 31, 29.5 Infralevator: 58, 55.2 Anterior urogenital triangle: 4, 3.8	NA	NA	16, 15	NA
Tokunaga et al. [35]		NA	513, 313–660^a^	80, 10–168^a^	Pelvic cavity abscess: 1, 5 Chylorrea: 1, 5 BO: 2, 11 Wound infection: 1, 5 Urinary disturbance: 1, 5	≥ II: 0	13, 8–29^a^	LAR: 5, 26.3 ISR: 7, 36.8 APR: 7, 36.8 LPND: Unilateral: 7, 36.8; Bilateral: 20, 63^*^	13.5, 3–24^a^	NA	0	NA
Williams et al. [36]		NA	450–310–635	400, 150–1000	UTI: 2, 40 Ileus: 3, 60 Ureteric stricture: 1, 20 Perineal WD/infection: 1, 20	I: 3, 60 II: 2, 40	9, 7–23.5	APE: 5, 100 LAR + AOR: 2, 40	NA	NA	NA	NA
Yamaguchi et al. [37]		Stage IV tumors; TPE; Synchronous/metachronous malignant lesions (within 5 years) other than carcinoma in situ; Conventional laparoscopic surgery	NA	NA	NA	NA	NA	LAR + LPND: 40, 51.3 ISR + LPND: 25, 32.1 APR + LPND: 13, 16.7	48, 19–112^a^	NA	NA	NA
Yang et al. [38]		Poor anal function; Distant metastasis; Tumor extending across the mid-line, beyond the ipsilateral LAM at the level of the anorectal ring and directly invades or adheres to adjacent organs or structure, according to the pretreatment MRI; Tumors occupying more than half of the bowel lumen	374.4^b^, 124.9^c^	321.7^b^, 124.9^c^	Overall: 7, 30.4% AL: 4, 17.4% Parastomal hernia: 1, 4.3% Acute UR: 2, 8.7%	I: 0 II 4, 17.3 III: 3, 13.1	11.4^b^, 9.1^c^	LAR + PELM: 23, 100	NA	NA	NA	NA
Yatabe et al. [39]		NA	690, 579–812	340, 165–815	Organ space SSI: 12, 20 Ileus: 9,15 UTI: 3, 5 AL: 2, 3	≧ II: 28, 48 (30 d) ≧ III: 14, 24 ≧ III: 9, 15 (90 d)	25, 21–34	TPE: 36, 61 APE: 16, 27 PPE: 7, 12	NA	NA	0	NA

Summary: Perioperative outcomes across 19 studies showed a wide range in operative time (median 280–690 min) and EBL (median 20–500 ml, range 0–2000 ml), with conversion rates remaining low at 1.8%. Urinary complications, AL and ileus were the most reported postoperative issues. CD ≥ III complications occurred in 18.7% of patients, while 30-day reoperation and readmission rates were 8.9% and 15.9%, respectively. LPND was the most frequently reported resected compartment, and postoperative LOS varied widely, with a median < 10 days in over half of the reported studies

*IQR* interquartile range, *EBL* estimated blood loss, *CD* Clavien-Dindo, *LOS* Length of stay, *LN* Lymph node, *NA* not available for retrieval, *nCRT* neoadjuvant chemoradiotherapy, *NART* neoadjuvant radiotherapy, *TPE* total pelvic exenteration, *LAM* levator ani muscle, *LL* lymphatic leak, *UR* urinary retention *AL* anastomotic leak, *WD* wound dehiscence, *SSI* surgical site infection, *UL* urinary leak, *BO* bowel obstruction, *PE* pulmonary embolism, *SVT* supraventricular tachycardia, *UTI* urinary tract infection, *30 d* within 30 days, *90 d* within 90 days, *TME* total mesorectal excision, *APER* abdominoperineal excision of the rectum, *LPND* lateral pelvic node dissection, *LAR* low anterior resection, *ISR* intersphincteric resection, *APR* abdoperineal resection, *PE* pelvic exenteration, *bTME* beyond total mesorectal excision, *AOR* additional organ resected, *TPE* total pelvic exenteration, *PPE* posterior pelvic exenteration, *RPLND* retroperitoneal lymph node dissection, *MVR* multivisceral resection, *ELAPE* extralevator abdoperineal excision, *APE* anterior pelvic resection, *PELM* partial excision of the levator ani muscle

^a^Range

^b^Mean

^c^Standard deviation

^*^Error in source data

### Oncological data

Eleven studies [6, 7, 20, 25, 26, 28, 29, 34, 36, 39] reported resection margin status, with an overall R0 rate of 92.6% (636/687 patients) and R1 or greater in 7.4% (51/687). CRM was assessed in nine studies [26, 28–30, 32, 33, 36–38], with negative margins achieved in 94.9% of patients (335/353).

Pathological staging was reported in 13 studies [6, 7, 20, 22, 26–28, 30, 32–34, 37, 38], with the most common pathological subcategories being ypT3 (n = 376, 52.4%), ypN0 (n = 403, 55.6%), and ypM0 (n = 84, 83.2%). The most frequently reported overall pathological stage was stage III (including IIIA, IIIB, and IIIC), comprising 44.2% of patients (n = 42), reported in three studies [30, 32, 38].

Perioperative data were available from all 19 studies. Apart from Tokunaga et al. [35] and Yamaguchi et al. [37], all studies included patients who predominantly underwent neoadjuvant therapy; in one study [30], omission of radiotherapy was an exclusion criterion. Adjuvant therapy reporting was less consistent, with two of eight relevant studies [28, 29] not specifying the treatment type.

Mortality outcomes were described in 13 studies [7, 22, 25–27, 29–32, 34–36, 39]. Two studies reported no deaths during the unspecified postoperative period [32, 34]. Six studies [7, 26, 27, 31, 35, 39] specifically reported 30-day mortality, with an overall rate of 0.8% (3/359 patients) (Table 5).

**Table 5:** Oncological data

First author		R classification (n, %)	CRM (n, %)	Pathological staging				Neoadjuvant therapy (n, %)	Adjuvant therapy (n, %)	Mortality (n, %)
				ypT (n, %)	ypN (n, %)	ypM (n, %)	Overall (n, %)			
Chang et al. [25]		R0: 11, 100^*^	NA	NA	NA	NA	NA	Overall: 6, 35 CTx: 3, 18 Concurrent CRT: 3, 12 CTx + SC RTx: 1, 6	NA	1, 6^b^ (FU)
Dibrito et al. [26]		R0: 78, 90.7 R1: 8, 9.3	neg: 78, 90.7 pos: 8, 9.3	T0: 4, 4.7 T1: 4, 4.7 T2: 12, 14 T3: 54, 68.2 T4: 1, 1.2 T4a: 5, 5.8 T4b: 6, 7	N0: 48, 55.8 N1: 1, 1.2 N1a: 11, 12.8 N1b: 9, 10.5 N1c: 5, 5.8 N2a: 8, 9.3 N2b: 4, 4.7	M0: 73, 84.9 M1: 4, 4.7 M1a: 7, 8.1 M1b: 2, 2.3	NA	Overall: 71, 82.5 TNT: 37, 43.0 SC RTx: 65, 75.6 LC CRT: 25, 29.1 CTx: 9, 10.5	CTx: 48, 55.8 None: 38, 44.2	1, 1.2 (30 d)
Ishizaki et al. [27]		NA	NA	T1: 3, 11.1 T2: 6, 22.2 T3: 15, 55.6 T4: 3, 11.1	N0: 9, 33.3 N1: 10, 37.0 N2: 8, 29.6	NA	NA	CTx: 27, 100^a^	NA	0 (30 d)
Jaganmurugan et al. [28]		R0: 5, 100	neg: 5, 100	T1: 1, 20 T4: 4, 80	N0: 1, 20 N1: 4, 80	M0: 4, 80 M1: 1, 20	NA	CRT: 4, 80 CTx + sequential CRT: 1, 20	3, 60	NA
Kazi et al. [29]		R0: 68, 92 R+: 6, 8.1	neg: 67, 91 pos: 7, 9.5	NA	NA	NA	NA	LC CRT: 37, 50 SC RTx: 1, 1.4 CRT + CTx: 13, 18 SC RTx + CTx; 22, 30 None: 1, 1.4	61, 82.4	5, 6.8 (FU)
Khan et al. [7]		R0: 156, 92.9	NA	T0: 22, 13.1 T1: 11, 6.5 T2: 27, 16.1 T3: 60, 35.7 T4: 48, 28.6	N0: 127, 75.6 N1: 24, 14.3 N2: 17, 10.1	NA	NA	CRT: 125, 74.4 (LC: 117, 92.9 SC: 9, 7.1)	CTx; 72, 42.9	2, 1.2 (30 d)
Kim et al. [22]		NA	NA	T0–T2: 31, 31.0 T3/T4: 69, 69.0	NA	NA	NA	CRT: 90, 90.0	NA	0 (FU)
Larach et al. [20]		R0: 23, 95.8 R1: 1, 4.2	NA	T3: 13, 61.9 T4: 7, 33.3 *is*^e^: 1, 4.8^c^	N0: 14, 66.7 N1: 5, 23.8 N2: 2, 9.5^c^	NA	NA	LC CRT: 21, 87.5^d^ CTx + CRT: 1, 4.1	CTx: 10, 47.6^c^	NA
Peacock et al. [30]		NA	neg: 40, 100	T0: 4, 11.1 T1: 2, 5.6 T2: 7, 19.4 T3: 22, 61.1 T4b: 1, 2.8^f^	N0: 17, 47.2 N1a: 9, 25 N1b: 3, 8.3 N2a: 2, 5.6 N2b: 5, 13.9^f^	NA	0: 4, 11.1 I: 4, 11.1 IIA: 9, 25 IIC: 1, 2.8 IIIA: 6, 16.7 IIIB: 5, 13.9 IIIC: 4, 11.1 IV: 3, 8.3^f^	Overall: 40, 100 CRT: 6, 15 CRT + CTx: 32, 80 CTx + RTx: 2, 5	NA	3, 7.5 (FU) 0 (90 d)
Piozzi et al. [6]	Lateral	R0: 27, 90.0 R1: 1, 3.3 R2: 2, 6.7	NA	T0: 1, 3.3 T1: 1, 3.3 T2: 8, 36.7 T3: 18, 60.0 T4: 2, 6.7	N0: 8, 26.7 N1: 15, 50.0 N2: 7, 23.6	NA	NA	Overall: 27, 90.0 SC CRT: 20, 66.7 LC CRT: 7, 23.3	NA	NA
	Longitudinal	R0: 83, 94.3 R1: 5, 5.9 R2: 0		T0: 4, 4.5 T1: 3, 3.4 T2: 24, 27.3 T3: 51, 58.0 T4: 6, 6.8	N0: 53, 60.2 N1: 31, 35.2 N2: 4, 4.5			Overall: 67, 76.1 SC CRT: 5, 5.7 LC CRT: 61, 69.3 CTx: 1, 1.1		
	Radial	R0: 17, 89.5 R1: 1, 5.3 R2: 1, 5.3		T2: 1, 5.3 T3: 6, 31.6 T4: 12, 63.2	N0: 11, 57.9 N1: 4, 21.1 N2: 4, 21.1			SC CRT: 17, 89.5		
Saqib et al. [31]		R0: 13, 100	NA	NA	NA	NA	NA	RTx: 13, 100	NA	4, 30.7 (FU) 0 (30 d)
Shin et al. [32]		NA	neg: 36, 100	T0/T1/T2: 11, 30.6 T3: 19, 52.8 T4a: 3, 8.3 T4b: 3, 8.3	N0: 11, 30.6 N1: 16, 44.4 N2: 9, 25	NA	≤ I: 5, 13.9 II: 5, 13.9 III: 18, 50 IV: 8, 22.2	CRT: 32, 88.9	CTx: 28, 90.3^g^	0 (POp)
Sieffert et al. [33]		NA	neg: 6, 100	T3: 4, 66.7 T4b: 2, 33.3	N0: 4, 66.7 N1b: 2, 33.3	M0: 5, 83.3 M1: 1, 16.7	NA	CRT: 6, 100	CTx: 5, 83	NA
Thorgersen et al. [34]		R0: 95, 90.5 R1: 10, 9.5	NA	T0: 11, 10 T1: 5, 5 T2: 20, 19 T3: 61, 58 T4a: 1, 1 T4b: 7, 7	N0: 52, 49 N1: 41, 39 N2: 9, 9	M0: 2, 2 M1: 2, 2	NA	SC RTx: 32, 31 LC RTx: 72, 69	NA	0 (POp)
Tokunaga et al. [35]		NA	NA	NA	NA	NA	NA	CRT/CTx: 7, 35 None: 13, 65	NA	0 (30 d)
Williams et al. [36]		R0: 4, 80 R1: 1, 20	neg: 4, 80 pos: 1, 20	NA	NA	NA	NA	CRT: 5, 100	CTx: 4, 80	1, 20 (FU)
Yamaguchi et al. [37]		NA	neg: 78, 100	T1: 2, 2.6 T2: 20, 25.6 T3: 46, 59 T4: 10, 12.8	N0: 33, 42.3 N1: 25, 32.1 N2: 20, 25.6	NA	NA	CRT: 6, 7.7	CTx: 40, 51.3	NA
Yang et al. [38]		NA	neg: 21, 91.3 pos: 2, 8.7	T0: 5, 21.7 T1: 4, 17.4 T2: 5, 21.7 T3: 7, 30.4 T4: 2, 8.7	N0: 15, 65.2 N1: 7, 30.4 N2: 1, 4.3	NA	I: 5, 21.7 II: 4, 17.4 III: 9, 39.1 IV: 5, 21.7	LC CRT: 23, 100	CTx: 19, 82.6	NA
Yatabe et al. [39]		RM0: 56, 95^h^ RM1: 3, 5	NA	NA	NA	NA	NA	CTx: 32, 54 RTx: 14, 24	NA	1, 2 (30 d)

Summary: Among the 11 studies reporting resection margins, the overall R0 rate was 92.6%, with CRM involvement identified in only 5.1% of patients. Pathological staging showed a predominance of ypT3 and ypN0 disease, with stage III variants being most common overall. Neoadjuvant therapy was administered in most studies, while adjuvant therapy was inconsistently reported. 30-day postoperative mortality was low at 0.8% across six studies

*CRM* Circumferential Resection Margin, *NA* not available for retrieval, *neg* negative, *pos* positive, *CRT* chemoradiotherapy, *CTx* chemotherapy, *RTx* radiotherapy, *FU* follow-up period, *SC* short-course, *LC* long-course, *FU* within follow-up period, *POp* postoperative period, *30 d* within 30 days postoperatively, *90 d* within 90 days postoperatively

^a^Chemotherapy regimen: FOLFOX: 8, 30.0%; XELOX: 15, 55.6%; SOX: 1, 3.7%; Others: 3, 11.1%

^b^Died within five months secondary to local and distant metastatic disease

^c^These oncological data were only reported for 21 patients who had rectal adenocarcinoma, rather than the 24 total patients

^d^These patients all had rectal cancer

^e^This patient had areas of adenocarcinoma in situ, and a simultaneous prostatic adenocarcinoma

^f^These pathological staging data were only reported for 36 patients with resections of the primary rectal cancer and concurrent lateral lymph node dissection, rather than the 40 total patients

^g^Among 31 patients who underwent curative surgery for advanced tumors

^h^RM0 defined as resection with margin of normal tissue from tumor edge, regardless of distance

^*^Error in source data

### Follow-up data

Most studies (11/14, 78.6%) reported a follow-up period of at least one year [6, 7, 22, 25, 26, 29–32, 37, 38]. Across these follow-up intervals, the overall recurrence rate was 24.9% (113/453 patients), based on 11 studies [7, 20, 25, 26, 28, 29, 31–33, 36, 38]. Local recurrence occurred in 7.6% of patients (61/804) across 14 studies [6, 7, 20, 22, 25, 26, 28–30, 32, 33, 35, 36, 38], while distant recurrence was reported in 19.2% (85/442) across nine studies [7, 20, 25, 26, 28–30, 36, 38]. Among six studies that detailed the site of distant recurrence [7, 26, 28, 32, 36, 38], the lungs were most involved, occurring in 10.5% of patients (34/323).

Survival outcomes were reported in nine studies [6, 7, 25, 26, 29, 32, 36–38], though with varied follow-up time points, making direct comparison difficult. One-year survival exceeded 90% in two studies [6, 7], while 3-year survival was over 80% in four studies [6, 7, 28, 40]. Five-year survival ranged from 74.9% to 95.4% [7, 32, 37]. The 3-year disease-free survival was 67.6% based on four studies [6, 7, 26, 38], and 3-year local recurrence-free survival was 87.2%, reported in three studies [6, 26, 38] (Table 6).

**Table 6:** Follow-up data

First author		Follow-up duration (months, med, IQR)	Overall recurrence (n, %)	Local recurrence (n, %)	Distant recurrence (n, %)	Overall survival (years: %)	Disease-free survival (years: %)	Local recurrence-free survival (years: %)
Chang et al. [25]		18, 1–73^a^	1, 6	1, 6	1, 6	NA	NA	6 months: 94
Dibrito et al. [26]		28.4	18, 20.9	Overall: 4, 5 Anastomosis: 2, 2 Pelvic lymph nodes: 2, 2 Mesenteric nodes: 1, 1	Overall: 14, 16 Liver: 7, 8 Lung: 10, 12 Adrenal: 1, 1 Central retroperitoneal nodes: 4, 5	3: 88.4 5: 74.9	3: 69.8	3: 93.9
Ishizaki et al. [27]		NA	NA	NA	NA	NA	NA	NA
Jaganmurugan et al. [28]		10	2, 40	0	Paraaortic lymph nodes and lungs: 2, 40	NA	NA	NA
Kazi et al. [29]		19, 12–47	23, 31	6, 8.1	16, 22	2: 91	2: 63.1	2: 87.7
Khan et al. [7]		34.0, 10.0–65.7	40, 23.8	15, 8.9	Overall: 35, 20.8 Lung: 14, 8.3 Liver: 12, 7.1 Brain: 4, 2.4 Peritoneum: 3, 1.8 Para-aortic lymph nodes: 2, 1.2 Other sites: 7, 4.2	1: 91.7 3: 82.1 5: 76.8	1: 84.0 3: 74.5 5: 69.2	NA
Kim et al. [22]		44.2^b^	NA	6, 6	NA	NA	NA	NA
Larach et al. [20]		10, 7–23.5	5, 20.8	0	5, 20.8	NA	NA	NA
Peacock et al. [30]		16, 5–22	NA	1, 2.5	7, 17.5	NA	NA	NA
Piozzi et al. [6]	Lateral	47, 31.5–66.5	NA	9, 30.0	NA	1: 96.6 3: 84.8	1: 78.7 3: 50.2	1: 85.8% 3: 68.8%
	Longitudinal			10, 11.4		1: 97.7 3: 86.9	1: 88.6 3: 58.7	1: 96.5% 3: 87.9%
	Radial			2, 10.5		1: 77.8 3: 55.0	1: 65.8 3: 59.8	1: 87.7% 3: NA
Saqib et al. [31]		21, 3–53^a^	3, 23.7	NA	NA	NA	NA	NA
Shin et al. [32]		30, 14.75–46	11, 35	1, 3	Overall: NA Lungs: 7, 23 Liver: 4, 13	5: 80	5: 54.6	5: 96.4
Sieffert et al. [33]		NA	2, 33	1, 17	1, 17	NA	NA	NA
Thorgersen et al. [34]		FU: 3 and 9 months	NA	NA	NA	NA	NA	NA
Tokunaga et al. [35]		NA	NA	NA	NA	NA	NA	NA
Williams et al. [36]		NA	2, 40	Pelvis: 1, 20	Overall: 2, 40 Retrocrural/supraclavicular nodes: 1, 20 Lung and femoral neck: 1, 20	2: 80	NA	1: 0 2: 40
Yamaguchi et al. [37]		41.6, 13.6–63.6^a^	NA	Lateral lymph node: 1, 1	NA	5: 95.4	NA	5: 98.6
Yang et al. [38]		44.1^b^	6, 26	Overall: 3, 13 Anastomosis site: 2, 9 Presacral site: 1, 4	Liver, lungs and paraaortic lymph nodes: 3, 13	3: 95	3: 72.4	3: 85.6
Yatabe et al. [39]		NA	NA	NA	NA	NA	NA	NA

Summary: Most studies reported a follow-up duration of ≥ one year, with an overall recurrence rate of 24.9%, including local recurrence in 7.6% and distant recurrence in 19.2%, most commonly in the lungs. Survival outcomes were variably reported, with 1-year overall survival > 90%, 3-year survival > 80%, and 5-year survival ranging from 74.9% to 95.4%. 3-year disease-free and local recurrence-free survival were 67.6% and 87.2%, respectively

*NA* not available for retrieval, *FU* follow-up

^a^Range

^b^Mean

### Quality assessment results

**Fig. 2:**
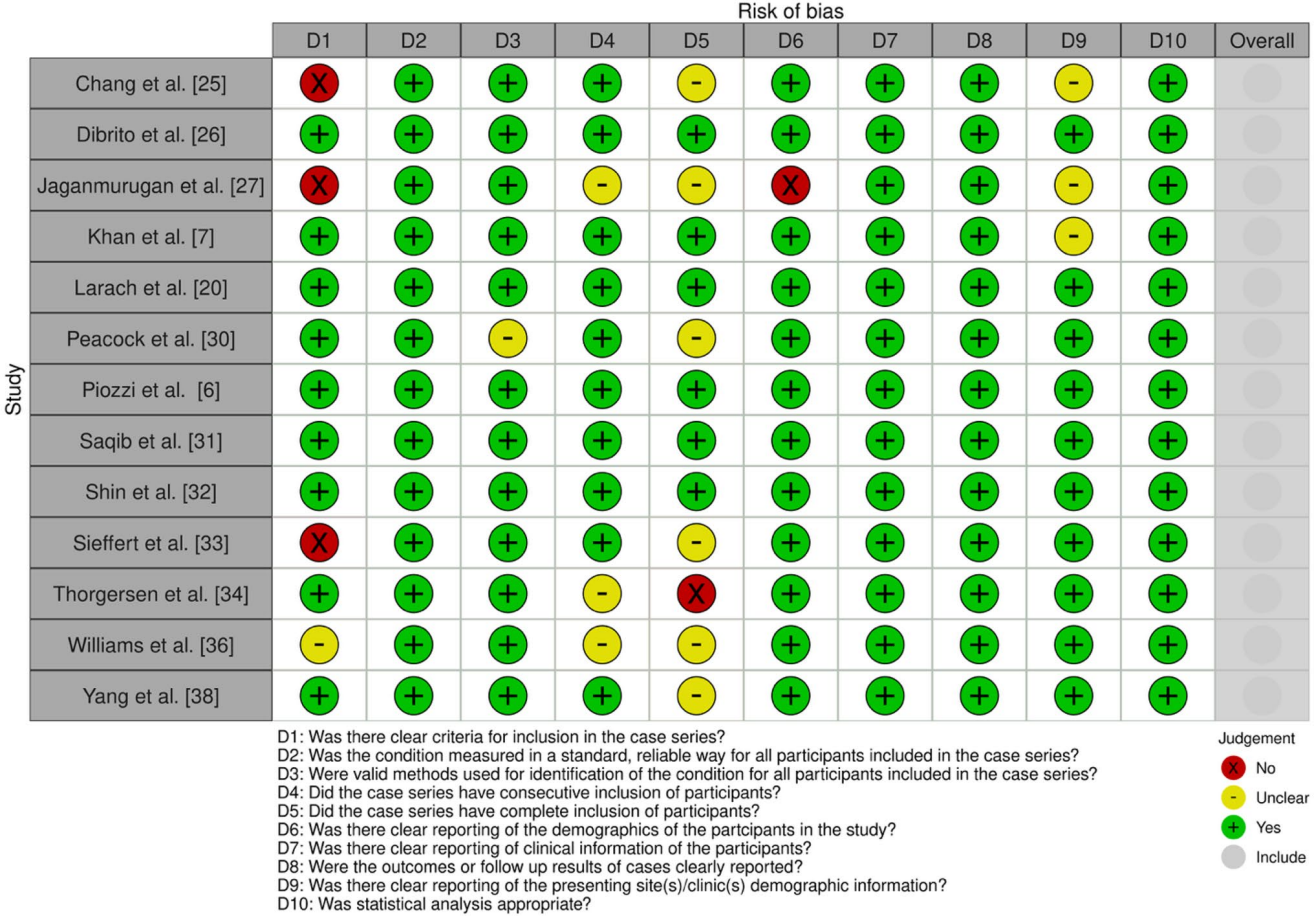
Quality assessment using the JBI Critical Appraisal Checklist for Case Series [23]. Plot created using robvis software [40]

**Fig. 3:**
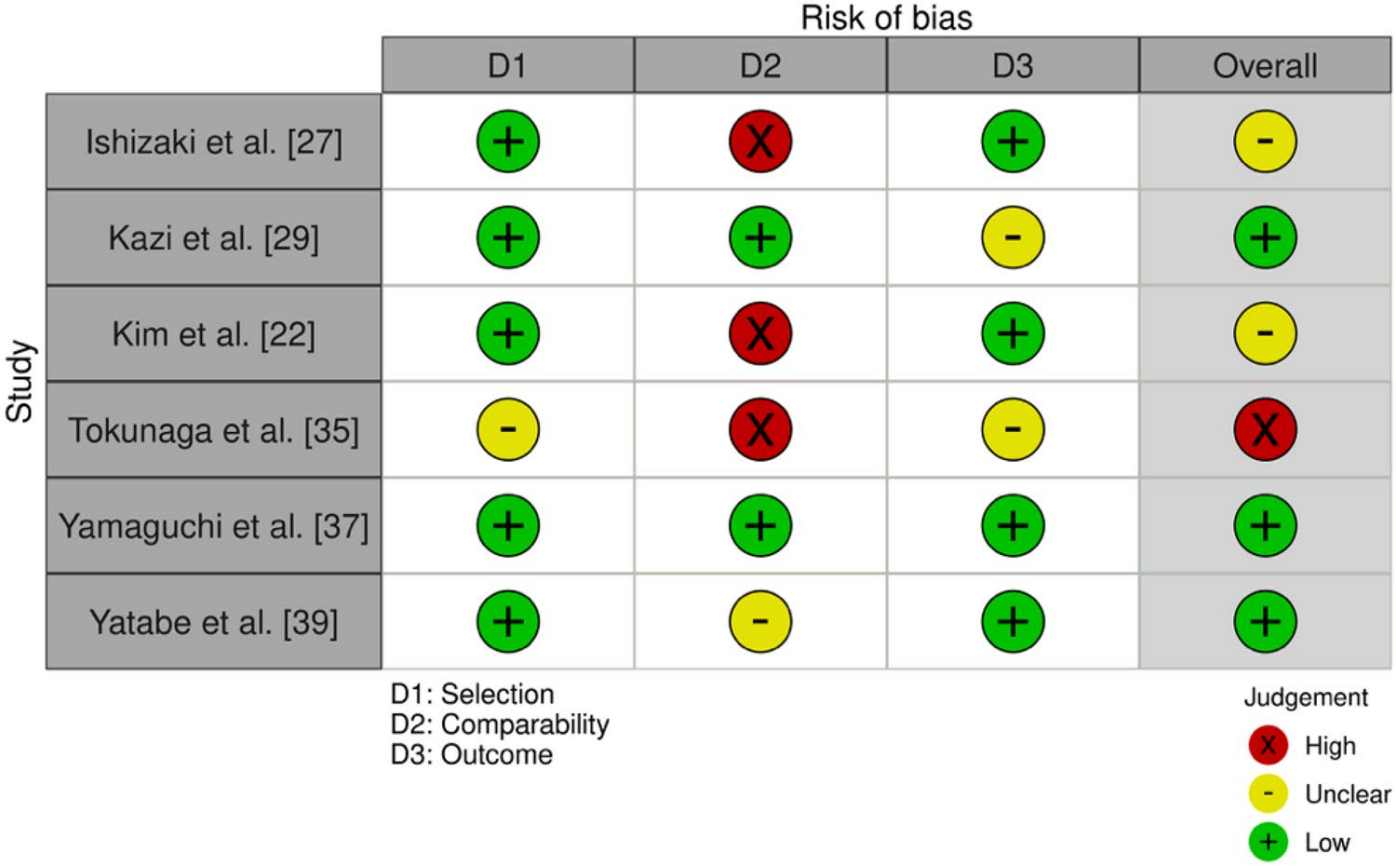
Quality assessment using Modified Newcastle–Ottawa Score for non-randomized studies [24]. Plot created using robvis software [40]

The retrieved studies were assessed using different approaches due to their study designs. The case series were of overall good quality when assessing individual domains. However, among the 6 cohort studies of largely moderate to low risk of bias, only 1 study, Tokunaga et al. [35], posed a severe risk due to the unclear nature of control population selection, lack of controlled variables such as age or sex, and a short follow-up period of only 30 days (Figs. 2 and 3.

## Discussion

bTME for locally advanced or recurrent anorectal cancer is a technically demanding but increasingly adopted approach. While open surgery remains the gold standard in many centers, this systematic review of 19 retrospective studies encompassing 1027 patients suggests that robotic bTME can achieve comparable oncological outcomes with potentially improved perioperative parameters.

Across the included studies, most patients were male, with BMIs in the 20–30 kg/m^2^ range and ASA scores predominantly of 2. Most cases were primary advanced rectal cancers, with a smaller proportion being recurrent. Notably, the R0 resection rate was consistently ≥ 90% (except in Williams et al. [36]), and CRM positivity rate was low at an overall average of 5.3%. These outcomes are in line with, and in some series better than, similar open surgery results, where CRM positivity rates are reported to be around 5% [41] and R0 rates typically fall in the range of 75–90% [42, 43]. This suggests that robotic techniques can achieve oncological resection quality comparable to conventional open approaches.

In terms of morbidity, robotic bTME was associated with an overall complication rate of 18.7% for ≥ CD III events. Common postoperative complications included ileus, urinary retention, wound infection, and anastomotic leak. While these rates are not insignificant, they fall within the expected range for open exenterative procedures, which are known to carry complication rates as high as 30–50%, particularly in the setting of multivisceral or lateral pelvic resections [44–46]. In fact, some series suggest that with improved visualization, robotic bTME may reduce specific complications such as bleeding or ureteric injury, especially when pelvic fibrosis or distorted anatomy is present [17, 20, 47].

Despite these promising findings, it is important to acknowledge the significant technical and logistical challenges associated with robotic bTME. Unlike conventional total mesorectal excision, bTME involves extended dissections that may include sacrectomy, lateral compartment dissection, or en-bloc urogenital or osseous resections. The robotic approach requires adaptation of technique and often coordination between multiple surgical teams (e.g. colorectal, urology, plastic reconstruction), particularly when simultaneous abdominal and perineal fields are used. Some centers have adopted dual-console or multi-quadrant docking techniques, but this adds to operative time and resource use [48].

Further, the learning curve for robotic bTME is also steep. Most surgeons will have developed proficiency in standard robotic TME before attempting extended resections. Literature suggests that up to 40–50 robotic TME cases may be needed to reach technical competency, with additional volume required for complex multivisceral procedures [49, 50]. Moreover, few centers perform these surgeries frequently enough to accumulate sufficient experience quickly. As such, case selection and centralization to high-volume units are likely essential for safe implementation.

Cost is another critical factor when evaluating the role of robotic bTME. Robotic platforms are expensive to acquire and maintain, and per-case costs are significantly higher than for open or laparoscopic techniques, particularly when additional staff training and longer operating times are considered. Whether these costs are offset by reductions in complications, faster recovery, or shorter length of stay remains uncertain and has not been formally evaluated in the context of bTME. While robotic TME is consistently more expensive than laparoscopic or open approaches, some economic models in prostatectomy suggest that centralizing such procedures in high-volume centers may help improve cost-efficiency [51, 52]. However, this potential benefit has not yet been demonstrated in the context of extended resections such as bTME, especially in the long-term with increasing surgeon proficiency.

An important but underexplored area in the current literature is functional outcomes following robotic bTME. Pelvic exenterative surgery is known to carry a high risk of urinary and sexual dysfunction due to the proximity of autonomic nerves, bladder structures, and the need for dissection in fibrotic or previously irradiated fields [53, 54]. Robotic technology may allow for more precise dissection, better nerve preservation, and less traction on pelvic sidewalls, which could theoretically improve postoperative function. However, none of the included studies in this review formally assessed functional outcomes, and this remains a gap that future studies must address. Quality of life (QoL) metrics, including return to continence, sexual activity, and long-term stoma burden, are essential in assessing the true value of robotic approaches in this context.

Another challenge highlighted by this review is the heterogeneity in how procedures are described and classified. Only a minority of studies employed structured classification systems for bTME resections, such as those proposed in the Beyond TME Consensus Statement [55] or the pelvic exenteration lexicon by Burns et al. [8]. The absence of standardized nomenclature makes it difficult to compare outcomes across centers or stratify results by case complexity. Moving forward, the adoption of agreed terminology for describing lateral compartment involvement, sacral resection level, and organ involvement will be essential to allow more meaningful comparisons across studies and the development of risk-adjusted benchmarking.

Moreover, while robotic bTME appears to offer some technical advantages over the open approach, current evidence is limited by the lack of prospective or comparative studies. All included studies in this review were retrospective case series, many from single centers, with inherent risks of selection bias and publication bias. Few studies provided detailed data on matched open controls, and almost none included long-term follow-up beyond 2–3 years. Given the low annual case volumes for these procedures, multicenter prospective registries or collaborative audits may be the most feasible means of generating higher-quality evidence.

## Conclusion

The current body of evidence supports the feasibility and early safety of robotic bTME in selected patients with locally advanced or recurrent rectal cancer. While oncological outcomes such as CRM and R0 resection rates appear comparable to open surgery, further prospective studies are needed to confirm these findings. The robotic approach shows promise, particularly in terms of visualization, access and instrument control in complex pelvic dissections, but widespread adoption will require addressing key imitations including cost, training, and the lack of standardized outcome reporting.

## Data Availability

No datasets were generated or analysed during the current study.
